# Deformability-Based Electrokinetic Particle Separation

**DOI:** 10.3390/mi7090170

**Published:** 2016-09-20

**Authors:** Teng Zhou, Li-Hsien Yeh, Feng-Chen Li, Benjamin Mauroy, Sang Woo Joo

**Affiliations:** 1 Mechanical and Electrical Engineering College, Hainan University, Haikou 570228, China; zhouteng@hainu.edu.cn; 2 School of Mechanical Engineering, Yeungnam University, Gyongsan 712-719, Korea; 3 Department of Chemical and Materials Engineering, National Yunlin University of Science and Technology, Yunlin 64002, Taiwan; lhyeh@yuntech.edu.tw; 4 School of Energy Science and Engineering, Harbin Institute of Technology, Harbin 150001, China; litchi@hit.edu.cn; 5 Laboratoire JA Dieudonné, UMR CNRS 7351, Université Côte d’Azur, Université de Nice Sophia Antipolis, Parc Valrose 06108 Nice Cedex 02, France; benjamin.mauroy@unice.fr

**Keywords:** arbitrary Lagrangian–Eulerian (ALE), dielectrophoresis, microfluidic, particle separation

## Abstract

Deformability is an effective property that can be used in the separation of colloidal particles and cells. In this study, a microfluidic device is proposed and tested numerically for the sorting of deformable particles of various degrees. The separation process is numerically investigated by a direct numerical simulation of the fluid–particle–electric field interactions with an arbitrary Lagrangian–Eulerian finite-element method. The separation performance is investigated with the shear modulus of particles, the strength of the applied electric field, and the design of the contracted microfluidic devices as the main parameters. The results show that the particles with different shear moduli take different shapes and trajectories when passing through a microchannel contraction, enabling the separation of particles based on their difference in deformability.

## 1. Introduction

The separation of small particles is one of the most important steps in many chemical and biological analyses [1,2,3,4,5,6]. Over the past decade, many microfluidic devices for the separation of particles have been developed, including passive and active types [7,8,9,10,11,12]. Passive methods incorporate the internal force and the fluid mechanism, such as hydrophoretic filtration [13,14], hydrodynamic filtration (HDF) [15,16,17], lateral displacement (DLD) [18,19,20], and inertial forces [3,6,21,22,23], while active methods involve electrophoresis or dielectrophoresis (DEP) [4,24,25,26,27,28,29,30], magnetophoresis [2,31,32,33], optical methods [34,35], and acoustophoresis [36]. DEP is a phenomenon in which a force is exerted on a dielectric particle when it is subjected to a non-uniform electric field. DEP has great advantages: it is label-free, based on simple instruments, and correlated to high selectivity and sensitivity. In association with new and efficient microfluidic devices [27], DEP has been widely used to manipulate various micro/nano-scale bio-entities, such as cells [37], bacteria [38], and viruses [39,40].

A number of physical or topological properties of cells or particles, including size, shape, and deformability, can be used for separation. Some microfluidic separation devices that use the deformability of the motioned object have been proposed and validated. They are based either on inertia [41], obstacles [42] or on the DLD method [19]. In a straight microchannel, Hur et al. [41] found that deformability affects the particle equilibrium position, and were able to enrich cells using inertial force, cells’ deformability, and size acting as biomarkers. Zhu et al. [42] proposed a microfluidic device that can sort elastic capsules according to their deformability, using a channel with a semi-cylindrical obstacle and a diffuser. Using three-dimensional immersed-boundary finite-element lattice-Boltzmann simulations, Krueger et al. [19] demonstrated that DLD devices can be used to perform the deformability-based separation of red blood cells (RBC). Lin et al. [37] separated cancer cells from leukocytes based on size and deformability using a microfluidic ratchet mechanism. The aforementioned separation methods based on the particle deformability are passive methods [19,37,41,42], and rely either on both size and deformability or on significant differences in deformability.

**Figure 1:**
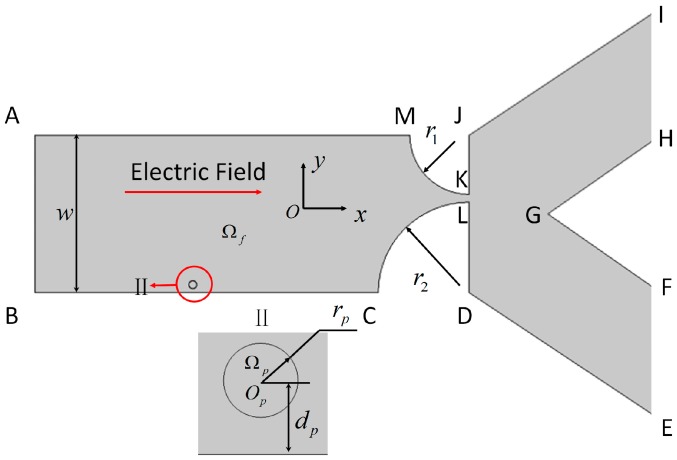
Electrokinetic motion of a deformable spherical particle of radius *r_p_* in a microfluidic chip with a contraction throat. *w*: width of the main channel. Widths of the channels with outlet IH and FE are identical. *r*_1_ and *r*_2_ are the radii of the two quad-circles, respectively; *d_p_* is the distance between the center of the spherical particle and the nearest channel wall.

Here we present a microfluidic device for the electric separation of particles based on their deformability, as shown in Figure 1. Separation is systematically investigated with numerical simulations. Fluid–structure interaction is simulated using finite elements and an arbitrary Lagrangian–Eulerian (ALE) method. The ALE method has been validated both experimentally and theoretically for rigid [5,12,13] and deformable [43,44] particles. The proposed microfluidic device is sensitive to the shear modulus of deformable particles, and is capable of separating particles with small differences in shear moduli. A parametric study is also conducted to optimize the performance of the proposed device.

The paper is organized as follows: Section 2 includes the theory for the deformable particle trajectory and separation mechanism. Section 3 presents the simulated results of the particle separation process and how the parametric studies—including the shear modulus, electric field intensity, and geometry parameters—affect the particle trajectory. Section 4 concludes the paper.

## 2. Formulation and Numerical Method

### 2.1. Mathematical Model

In this study, we consider a two-dimensional (2D) channel that consists of a uniform inlet section with a converging-expansion part, and two uniform outlet sections. This geometry is used to study electrokinetic particle translation, as shown in Figure 1. The contraction part of the converging-expansion channel is generated by two quad-circles with different radii. The two outlet sections are designed to sort particles with sufficiently different trajectories into two groups. A circular particle with radius *r_p_* located at a distance *d_p_* from the channel wall is shown in the magnified view below. An electric potential is applied externally from inlet AB to grounded outlets IH and FE to an incompressible Newtonian fluid domain Ω*_f_*. The electric field *E*, generated in the domain, induces the electrokinetic motion of a hyperelastic particle Ω*_p_* suspended in the fluid. Because the electric double layer (EDL) thicknesses adjacent to the charged particle and the channel wall are very thin in comparison to the particle radius and the channel widths, the thin-EDL approximation is applied. The electrical potential φ in the domain Ω*_f_* can be described by the Laplace equation,

∇^2^φ = 0 in Ω*_f_* (1)

The local electric field *E* can be calculated from the electric potential φ by

*E* = −∇φ in Ω*_f_* (2)

Because a potential shift is applied across the microfluidic chip, the boundary conditions for φ on the entrance and exits of the microchannel are

φ = φ_0_ on AB (3)

and

φ = 0 on IH and EF
(4)

Solid boundaries—including the channel wall (Г*_w_*) and particle surface (Г*_p_*)—are electrically insulating, yielding

*n*·∇φ = 0 on Г*_w_* and Г*_p_* (5)

where *n* is the unit outward normal vector.

The Reynolds number in the microchannel is very small, so that the conservation of momentum and mass can be described by the Stokes and the continuity equations:

(6) $$\rho_{f}\frac{\partial u}{\partial t}=\nabla \cdot [-pI+\mu (\nabla u+\nabla u^{T})]\;\mathrm{in}\Omega_{f}$$

and

∇·*u* = 0 in Ω*_f_* (7)

where ρ*_f_* and µ are the density and the viscosity of the fluid, respectively; *u* is the velocity vector; *p* is the hydrodynamic pressure; *I* is the unit tensor; and ∇*u^T^* is the transpose of the velocity gradient ∇*u*. An open boundary condition is specified at the inlet and the outlets:

∇·[−*p*I + µ(∇*u* + ∇*u^T^*)]= 0 on AB, IH and EF (8)

The Smoluchowski slip boundary condition for Newtonian electroosmotic flow (EOF) is applied on the charged channel wall:

(9) $$u=u_{w}=\frac{\varepsilon_{f}\zeta_{w}}{\mu }(I-nn)\cdot \nabla \phi \;\mathrm{on}{Г}_{w}$$

where *u_w_* is the fluid velocity on the channel wall, and ε*_f_* and ζ*_w_* are, respectively, the fluid permittivity and the zeta potential of the channel wall.

The velocity *u_p_* on the particle consists two parts: (i) the Smoluchowski slip velocity arising from the particle surface charge; and (ii) the velocity of the particle motion. The boundary condition on the particle surface is then

(10) $$u=u_{p}=\frac{\varepsilon_{f}\zeta_{w}}{\mu }(I-nn)\cdot \nabla \phi +\frac{\partial S}{\partial t}\;\mathrm{on}{Г}_{p}$$

where ζ*_p_* is the zeta potential of the particle and *S* is the displacement of the deformable particle caused by the particle deformation and movement, governed by

(11) $$\rho_{p}\frac{\partial^{2}S}{\partial t^{2}}-\nabla \cdot \sigma (S)=0\;\mathrm{in}\Omega_{p}$$

Here ρ*_p_* is the density of the deformable particle, and σ(*S*) is the Cauchy stress in the solid phase, which is a function of the displacement of the particle. In the following simulations, the particle is considered as an incompressible neo-Hookean material, described by a strain energy density function [43].

The force on the particle–fluid interface consists of hydrodynamic and electrokinetic stresses:

σ*_p_·n_p_ = *σ*_f_·n_f_ + *σ*_E_·n_f_* (12)

σ*_f_ = −pI* + µ(∇*u* + ∇*u^T^*) (13)

(14) $$\sigma_{E}=\varepsilon_{f}EE-\frac{1}{2}\varepsilon_{f}(E\cdot E)I$$

where σ*_p_*, σ*_f_*, and σ*_E_* are, respectively, the total stress tensor on the particle surface, the hydrodynamic stress tensor, and the Maxwell stress tensor, respectively.

### 2.2. Numerical Method and Code Validation

The above system is solved numerically using the commercial finite element package COMSOL (Version 4.3a, COMSOL Group, Stockholm, Sweden) coupled with MATLAB (Version 8.2, MathWorks Inc., Natick, MA, USA), operated in a high-performance cluster. The coupled system of hydrodynamic, electrical field, and particle mechanics is solved simultaneously. As we are using the ALE method, the mesh is deformed in order to follow the motion of the particle, and the mesh quality decreases progressively when the particle progresses into the microfluidic device. When the mesh quality falls below 0.7 (out of a maximum 1.0) [1,6,13], the domain is re-meshed with the current particle position, the solution is mapped to the new mesh, and the computation is restarted. The previous step is repeated each time the mesh quality falls below 0.7.

**Figure 2:**
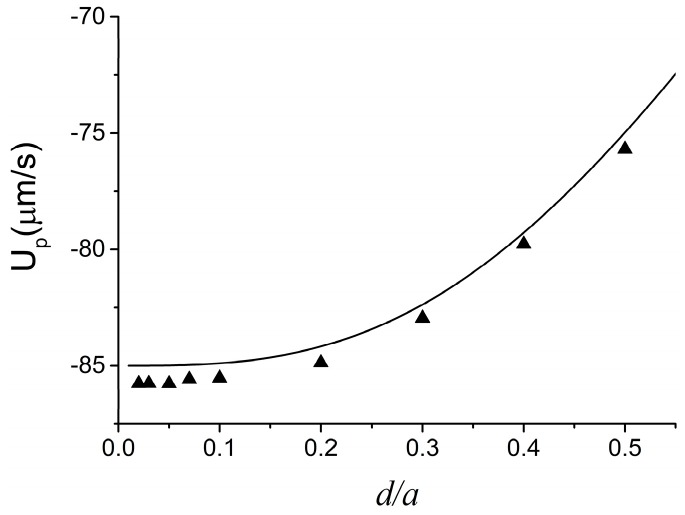
Velocity of a rigid sphere translating along the axis of a cylindrical tube as a function of the ratio between the diameter *d* of the sphere and diameter *a* of the channel. The solid line and triangle symbols represent the analytical solution of Keh and Anderson [45] and the numerical results from the present model, respectively.

To validate the present method, we compare its predictions with the analytical result of Keh and Anderson [45] for the electrophoresis of a rigid spherical particle of diameter *d* along the axis of an infinite long tube of diameter *a*. Under the conditions of thin EDL and negligible DEP force, the approximate analytical solution for the electrophoretic velocity of a spherical particle (*U_p_*) is

(15) $$U_{p}=\left[1-1.28987{\left(\frac{d}{a}\right)}^{3}+1.89632{\left(\frac{d}{a}\right)}^{5}-1.02780{\left(\frac{d}{a}\right)}^{6}\right]\left(1-\frac{\zeta_{w}}{\zeta_{p}}\right)U_{0}$$

where *U*_0_ = ε*_f_*ζ*_p_E_z/_*µ is the Smoluchowski velocity, with *E_z_* being the axial strength of the external electric field in the absence of particle. In the benchmark, *E_z_* = 30 kV, ζ*_w_* = 60 mV, ζ*_p_* = 20 mV, ε*_f_* = 7.08 × 10^−10^ F/m, ρ*_f_* = 1000 kg/m^3^, and µ = 0.001 Pa·s. To simulate a rigid particle, we used a very large value for *G* (*G* = 2000 Pa). As shown in Figure 2, the numerical predictions for *U_p_* using our method (symbols) are in good agreement with the analytical solution of Keh and Anderson [45] (solid line).

## 3. Results and Discussion

In the cases reported here, the geometric parameters are set to *w* = 200 µm, *r_p_* = 10 µm, and *d_p_* = 10 µm, and the physical properties of the aqueous solution are ρ*_f_* = 1000 kg/m^3^, µ = 0.001 Pa·s, and ε*_f_* = 7.08 × 10^−10^ F/m. The density and the permittivity of the neutrally buoyant deformable particle are assumed to be identical to those of the solution, ρ*_p_* = 1000 kg/m^3^ and ε*_p_* = 7.08 × 10^−10^ F/m. The zeta potentials on the channel wall (ζ*_w_*) and particle (ζ*_p_*) are set to −60 mV and 20 mV, respectively; the particle is to move from left to right.

In this section, the separation process is presented, after which a parametric study is performed to investigate the effects of shear modulus, electric field intensity, and geometrical parameters (*r*_1_ and *r*_2_) on the particle separation process.

### 3.1. The Separation Process

**Figure 3:**
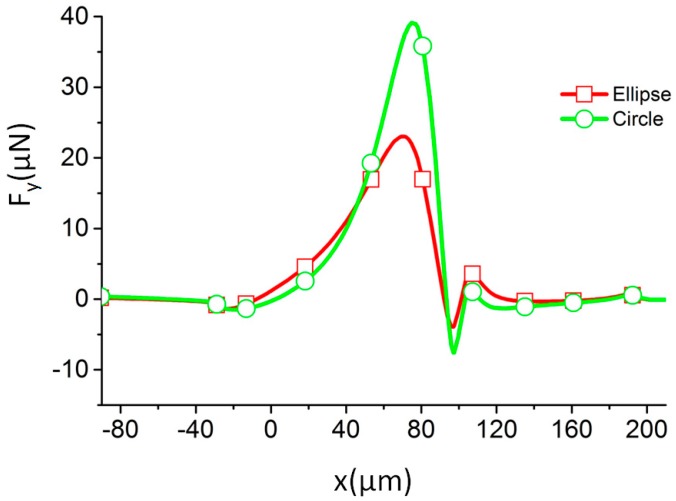
The spanwise component of dielectrophoretic (DEP) force along the channel for particles with two different shapes (identical volumes) with *r*_1_ = 60 µm, *r*_2_ = 120 µm. The radius of the circular particle is 5 µm, while the lengths of the major and minor axis of the ellipse are 6.25 and 4 µm, respectively.

The separation of dissimilar particles can be achieved by making their trajectories different. In this study, we chose to discriminate the particles using their shear modulus *G* as a marker. Particles with different shear moduli reach different deformations and shapes, even if their volumes are identical. Due to the difference in their shapes, the DEP force on the particles—which tends to push the particle away from the streamlines—is different, even if their location and surrounding electric field are identical. To illustrate that the DEP force on the particles depends on the particle shape, we calculated the DEP force in the spanwise direction for two different particles with the same volume: one with circular shape and one with elliptical shape. The distances from the centers of the particles to the wall were also set to be identical. In order to demonstrate the sole role of DEP forces on the two different particles, they are first fixed in space. Figure 3 shows that the force on the circular particle is larger than the force on the elliptical one. It is then expected that the circular particle will be pushed farther toward the center than the elliptical one. This also implies that the DEP focusing can be weakened by the particle deformation. In this way, particles with different shear moduli can be separated due to a difference in their deformations.

**Figure 4:**
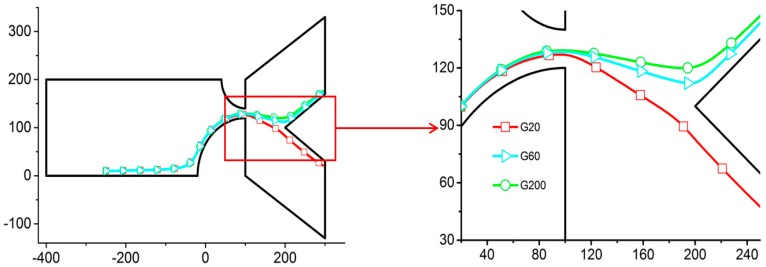
Trajectories of particles with different shear moduli *G* with *r*_1_ = 60 µm, *r*_2_ = 120 µm, and the strength of the axial electric field in the channel *E* = 30 V/m. An enlarged view of the contraction region is on the right. G*N*: shear modulus of the particle of *N* Pa. (axis in μm).

Figure 4 shows different trajectories for particles that exhibit only a difference in shear moduli. Shear moduli of 20, 40, 60, 80, 100, and 200 Pa are simulated, but only three cases are presented here for the sake of clarity. With different deformation and shape, the particles experience different hydrodynamic and Maxwell stresses, resulting in different particle trajectories for particles with different shear moduli.

**Figure 5:**
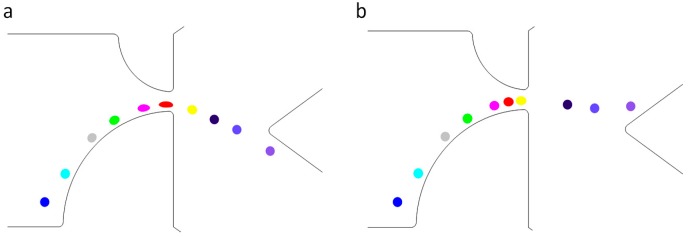
Time trace of a deformable particle passing through the contraction with the shear modulus (**a**) *G =* 20 Pa and (**b**) *G =* 200 Pa while *r*_1_ = 60 µm, *r*_2_ = 120 µm, and *E* = 30 V/m. The positions from left to right represent time lapse of 0, 25, 40, 46, 51, 53.5, 55, 60, and 80 ms.

**Figure 6:**
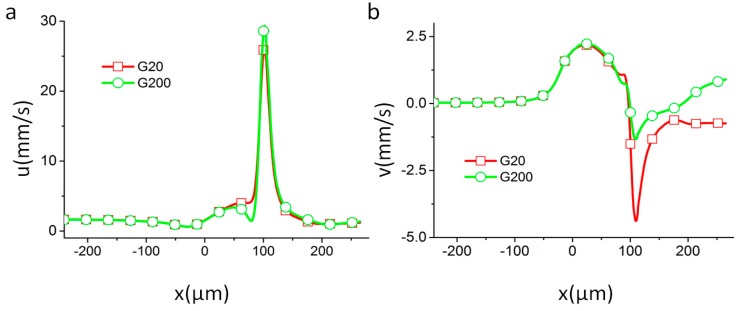
Velocity components of particles with different shear moduli in motion. (**a**) *u*, main flow direction; (**b**) *v*, orthogonal to main flow direction. Here *r*_1_ = 60 µm, *r*_2_ = 120 µm, and the electric field intensity in the channel is *E* = 30 V/m.

In Figure 5, two different particles with *G =* 20 and 200 Pa start from the same location. As the channel width decreases, the electric field intensity increases. At the beginning, the electric field is too weak to deform the particles, as between the first and third positions in Figure 5. The motion of the two particles is thus almost identical. The streamwise and spanwise components of the velocity vectors (shown in Figure 6) are also almost identical. As the particles progress through the channel, the electrical stress on the particle increases, inducing the particle with the lowest shear modulus to deform. In the fourth position in Figure 5, the electric field causes the particle with *G =* 20 Pa to deform, while it has little effect on the one with *G =* 200 Pa. Due to the difference in their shapes, the total forces on the two particles are different, as shown in Figure 3. Accordingly, the velocity components (shown in Figure 6) show conspicuous difference between particles with *G =* 20 Pa and *G =* 200 Pa. The spanwise components in particular show a negative value for *G =* 20 Pa, while that for *G =* 200 Pa has a positive value. The particles’ trajectories are fully separated upon reaching the channel constriction, where the electric field is the strongest.

### 3.2. Effect of Shear Modulus

The effect of particle compliance is studied by varying the shear modulus *G*. Considering that many engineering materials have shear modulus in the MPa or GPa range, most cells are in the kPa range, and some artificial liposomes can be as small as on the order of unity in Pa, a wide range of *G* was used in the experiment. Here we only report the results for *G* = 20 to 200 Pa because for the electric potential used, it is sufficiently wide to exhibit representative particle behaviors ranging from compliant to rigid. Between 20 and 60 Pa, the electric field intensity is strong enough to cause the particle to deform. The trajectories are not very different up to the contraction. As the particles leave the contraction and reach the expansion channel, the differences become notable. Due to the elasticity, the particle tends to recover its original shape as the electrical stress fades away. This relaxation process is slow, however, and the differences in trajectories persist. For this reason, we can separate particles based on their shear moduli with difference between their trajectories in the tested configuration, as shown in Figure 4.

### 3.3. Effect of External Electric Field

**Figure 7:**
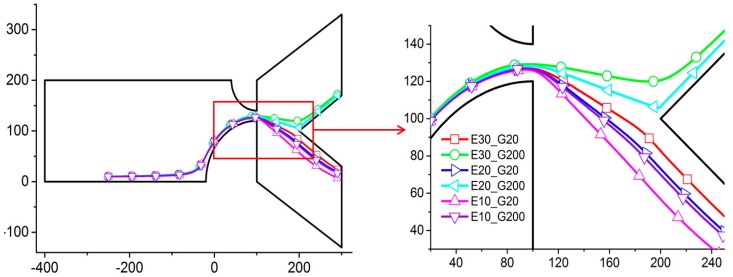
Trajectories of a particle for various combinations of the shear modulus *G* and strength of the axial electric field in the channel *E_x_* with *r*_1_ = 60 µm and *r*_2_ = 120 µm. On the right is an enlargement of the contraction region. E*i_*G*j* stands for the electric field strength in the channel at *i* V/m and the shear modulus of the particle at *j* Pa. Axes are in μm.

The effect of the electric field intensity is studied by varying its amplitude in the straight channel by adjusting the electric potential between the inlet and outlet, before the contraction channel to 20, 30, and 40 V/m. In order to show the clear separation performance, the particles with shear moduli *G* = 20 and 200 Pa are used (Figure 7). The result shows that particles with the same shear modulus move nearer to the upper wall when the electric field intensity increases. In Figure 7, the trajectories of particles with *G* = 200 Pa are the first, second, and fifth lines from top to bottom, with electric field intensities *E* = 30, 20, and 10 V/m. The difference in the trajectories for different *G* increases when the electric field intensity increases. In order to amplify the difference, high electric field intensities are needed. In practice, however, using high electric field intensity might not be possible, depending on the nature of the particles (e.g., biological particles). The present result shows that *E_x_* = 20 V/m is sufficient to separate the particles (Figure 7). The differences in the trajectories are, however, already very significant with *E* = 20 V/m, and such a reasonable electric field intensity should be sufficient for any actual applications [25,26,27].

### 3.4. Effect of Geometrical Parameters of the Contraction Region: r_1_ and r_2_

**Figure 8:**
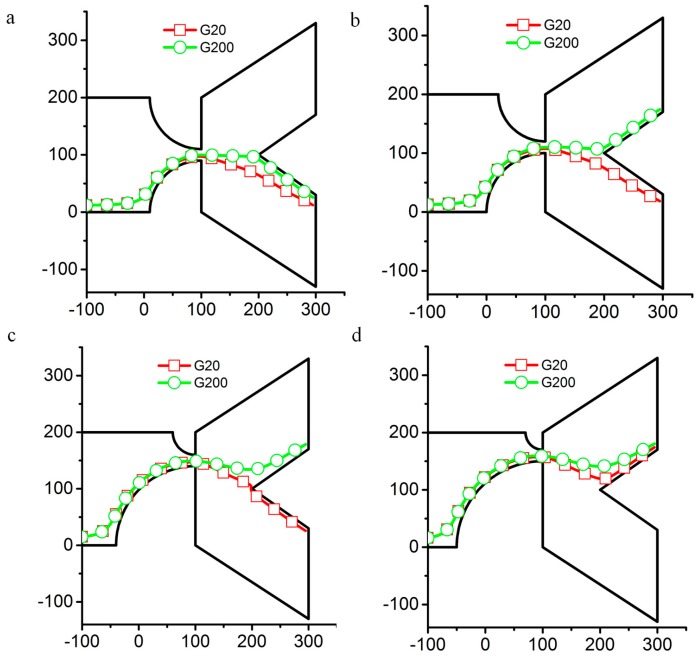
Trajectories of particles with two different shear moduli *G* for various values of *r*_1_ and *r*_2_ when the axial electric field in the channel is *E* = 30 V/m. (**a**) *r*_1_ = 90 µm and *r*_2_ = 90 µm; (**b**) *r*_1_ = 80 µm and *r*_2_ = 100 µm; (**c**) *r*_1_ = 40 µm and *r*_2_ = 140 µm; (**d**) *r*_1_ = 30 µm and *r*_2_ = 150 µm. Axes are in μm.

The geometrical parameters *r*_1_ and *r*_2_ can be used to control the particle trajectory (Figure 8). Due to the repelling force on the channel wall, the particle cannot stay very close to the channel wall in the contraction. The starting point of the particle is set to very close to the downward wall of the expansion channel. As the particle moves toward the contraction channel, the particle gradually moves away from the wall. There is thus a non-negligible distance between the particle and the channel wall. The particle position will be adjusted by the channel wall, composed of two circles. Then, changing the size of *r*_1_ and *r*_2_ can be used to control the outlet as the particle leaves the channel.

The particles with different shear moduli still have different trajectories, even when the sizes of *r*_1_ and *r*_2_ are varied. In Figure 8a, the particles with shear moduli *G* = 20 and 200 Pa all exit from the downward outlet with *r*_1_ = 90 µm and *r*_2_ = 90 µm. The figure shows that the repelling force is not strong enough to push the particle up to the centerline of the channel. In order to separate the particles, the sizes of *r*_1_ and *r*_2_ should be adjusted in order to make the particle with 200 Pa shear modulus cross the centerline and to keep the particle with 20 Pa shear modulus below the centerline. After we reduced *r*_1_ to 80 μm and increased *r*_2_ to 100 μm, the particle with 200 Pa shear modulus crosses the centerline and exits through the upper outlet, while the particle with 20 Pa shear modulus exits through the downward outlet (Figure 8b). We then sweep the sizes of *r*_1_ and *r*_2_ in 10 μm increments. When *r*_1_ = 40 µm and *r*_2_ = 140 µm, the particles with shear moduli *G* = 20 and 200 Pa can still be separated in the tested configuration. However, the particles all leave the channel through the upper outlet when *r*_1_ = 30 µm and *r*_2_ = 150 µm. This method shows that we can count on a 40 μm tolerance for the design of *r*_1_ and *r*_2_.

## 4. Conclusions

An electrokinetic microfluidic device for particle separation is designed and analyzed using an ALE-based finite elements computation. Separation is controlled with the particles’ shear modulus, which affects the particles’ deformation, in turn affecting the particles’ trajectories. The present study suggests that if the microfluidic device is properly designed, shear modulus is an effective separation marker for deformable particles. Furthermore, the proposed design exhibits a reasonable tolerance, which might ease any fabrication process. Additionally, we showed that low electric field intensities can be used without reducing separation efficiency, which allows the safeguarding of fragile particles, such as biological cells. Finally, we showed that geometrical parameters of the contraction channel provide flexibility in the design of the device proposed.
